# Shared signaling pathways and comprehensive therapeutic approaches among diabetes complications

**DOI:** 10.3389/fmed.2024.1497750

**Published:** 2025-01-08

**Authors:** Moein Ebrahimi, Hamid Ahmadieh, Mozhgan Rezaei Kanavi, Sare Safi, Saeed Alipour-Parsa, Soroor Advani, Christine M. Sorenson, Nader Sheibani

**Affiliations:** ^1^School of Medicine, Shahid Beheshti University of Medical Sciences, Tehran, Iran; ^2^Ophthalmic Research Center, Research Institute for Ophthalmology and Vision Science, Shahid Beheshti University of Medical Sciences, Tehran, Iran; ^3^Ocular Tissue Engineering Research Center, Research Institute for Ophthalmology and Vision Science, Shahid Beheshti University of Medical Sciences, Tehran, Iran; ^4^Ophthalmic Epidemiology Research Center, Research Institute for Ophthalmology and Vision Science, Shahid Beheshti University of Medical Sciences, Tehran, Iran; ^5^Cardiovascular Research Center, Shahid Modarres Hospital, Shahid Beheshti University of Medical Sciences, Tehran, Iran; ^6^Neurology Department, Shohada Tajrish Hospital, Shahid-Beheshti University of Medical Sciences, Tehran, Iran; ^7^Department of Pediatrics, University of Wisconsin School of Medicine and Public Health, Madison, WI, United States; ^8^Department of Ophthalmology and Visual Sciences, University of Wisconsin School of Medicine and Public Health, Madison, WI, United States

**Keywords:** diabetic retinopathy, diabetic nephropathy, diabetic neuropathy, diabetic cardiomyopathy, signal transduction, therapeutic targeting, metabolic dysregulation

## Abstract

The growing global prevalence of diabetes mellitus (DM), along with its associated complications, continues to rise. When clinically detected most DM complications are irreversible. It is therefore crucial to detect and address these complications early and systematically in order to improve patient care and outcomes. The current clinical practice often prioritizes DM complications by addressing one complication while overlooking others that could occur. It is proposed that the commonly targeted cell types including vascular cells, immune cells, glial cells, and fibroblasts that mediate DM complications, might share early responses to diabetes. In addition, the impact of one complication could be influenced by other complications. Recognizing and focusing on the shared early responses among DM complications, and the impacted cellular constituents, will allow to simultaneously address all DM-related complications and limit adverse treatment impacts. This review explores the current understanding of shared pathological signaling mechanisms among DM complications and recognizes new concepts that will benefit from further investigation in both basic and clinical settings. The ultimate goal is to develop more comprehensive treatment strategies, which effectively impact DM complications in multiple organs and improve patient care and outcomes.

## 1 Introduction

In 2021, 521 million individuals were living with diabetes mellitus (DM). It is predicted that by 2050, this figure will rise to over 1.3 billion individuals (1). The rise in the number of persons with DM, in addition to their improved life expectancy, has been associated with growing trends in DM complications in recent years (2, 3). Despite the rising DM prevalence and changing patterns of its consequences, curative strategies are still lacking. This is further impacted by the lack of a comprehensive understating of DM complications and their cellular constituents. Thus, preventing the simultaneous occurrence of DM complications, involving multiple targets, is crucial to improve patient care and outcomes.

The most challenging DM complications are traditionally defined as microvascular and macrovascular, which are classified into multiple categories including retinopathy, nephropathy, neuropathy, and cardiovascular disease. This classification is convenient at the clinical level, which provides a broad perspective of the comorbidities associated with DM and can be pragmatically translated into prevention and management recommendations. Early diagnosis of these complications is mainly based on vascular pathologies noted in a subset of organs (4, 5). In addition, DM affects almost every organ system beyond their vascular beds, with further damage that does not strictly fall into the artificial tissue-specific micro- and macro-vascular classification. In recent years, there have been challenging discussions regarding the temporal occurrence of DM-associated pathologies, such as retinal neurodegeneration and vasculopathies (6). These pathologies are highly intertwined and very challenging to unveil their hierarchies without the knowledge of what their cellular constituents are and how they temporally and spatially respond to diabetes.

The DM complications are linked in at least three ways and should not be studied in isolation. First, DM affects all organs simultaneously allowing one to examine each organ based on other organs and make a more accurate evaluation of DM’s impact on the specific integrity and function of other organs. When a DM complication occurs, the risk of incidence and progression of other complications rises (7). Second, organs interact with one another through systemic circulation. Renal failure for instance is the inability of the kidney to eliminate toxins from the body. Thus, even in non-diabetic renal diseases, uremic toxins resulting from chronic kidney disease, could influence retinal neurons (8). In addition, diabetic nephropathy increases the risk of developing diabetic retinopathy in diabetic patients (9), and retinal vascular density decreases in patients with chronic heart failure (10). Third, all organs require a blood supply provided by systemic circulation. However, tissue-specific variation in hemodynamics, vascular architecture, and cellular constituents could lead to organ-specific clinical presentations that are unique. Thus, the involvement of the vascular system could lead to a spectrum of pathological consequences in different target organs, whose simultaneous studies could provide a broader prospective of the systematic impact of DM and a broader impact on mitigation of its complications.

**Figure 1:**
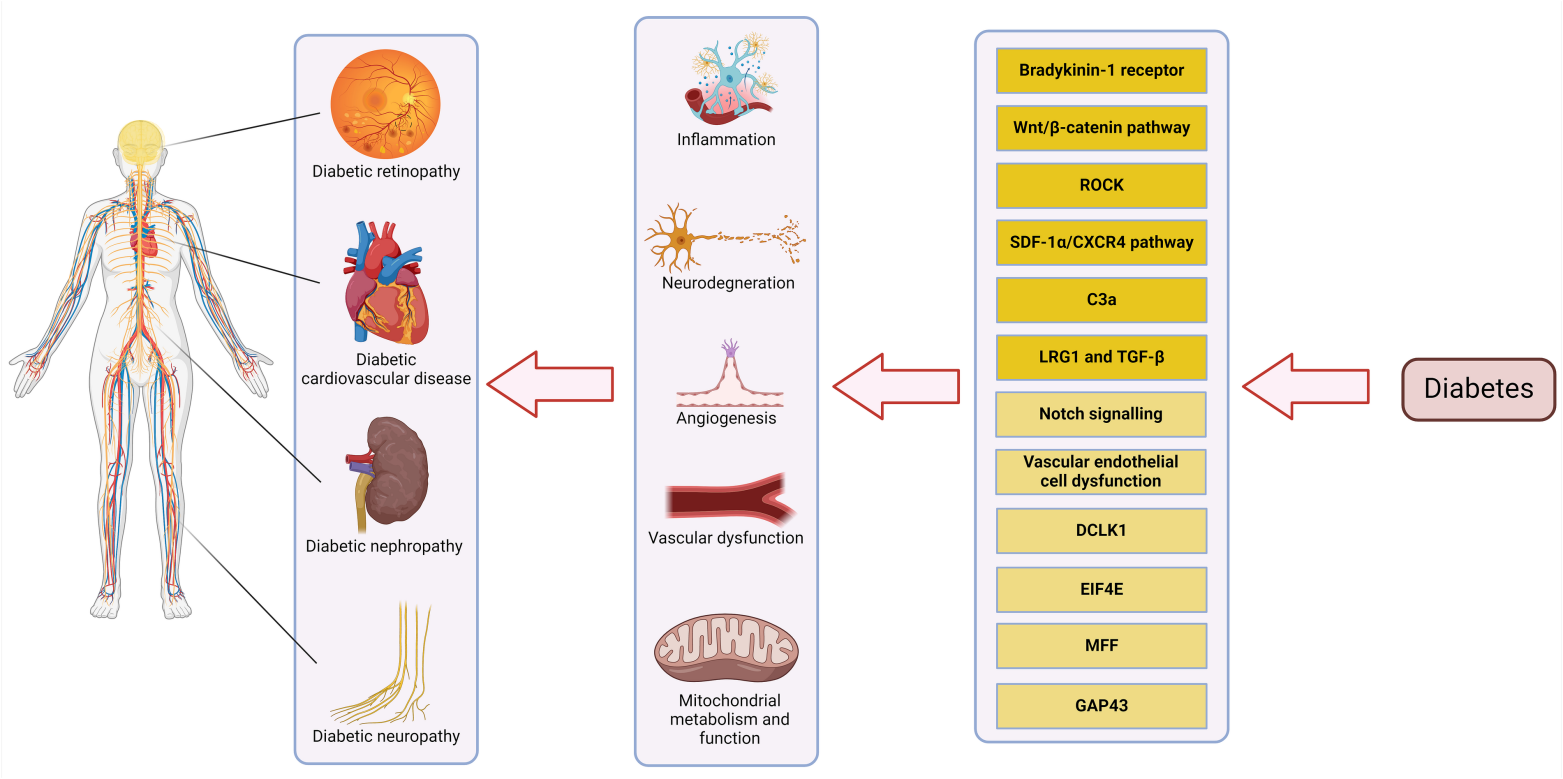
Pathways involved in DM complications. Pathways with completely clarified roles are highlighted with dark yellow and those which need more studies are highlighted with light yellow. Created with biorender.com.

Understanding the pathophysiology of DM complications at the systems level will aid in the discovery of additional methods of screening for early detection of these complications and offer treatment options that more comprehensively address the pathophysiology of disease. Here we review and discuss some of the shared pathologies among DM complications in various target organs and their cellular constituents, including the retina, kidney, heart, and peripheral nerves that could benefit from further basic and clinical research (Figure 1). The simultaneous extension of these studies to other target sites will provide broader perspective regarding DM systematic nature. This knowledge will aid in a more effective diagnosis and treatment for the disease.

## 2 The need for an interdisciplinary approach for diagnosis and treatment of DM complications

**Figure 2:**
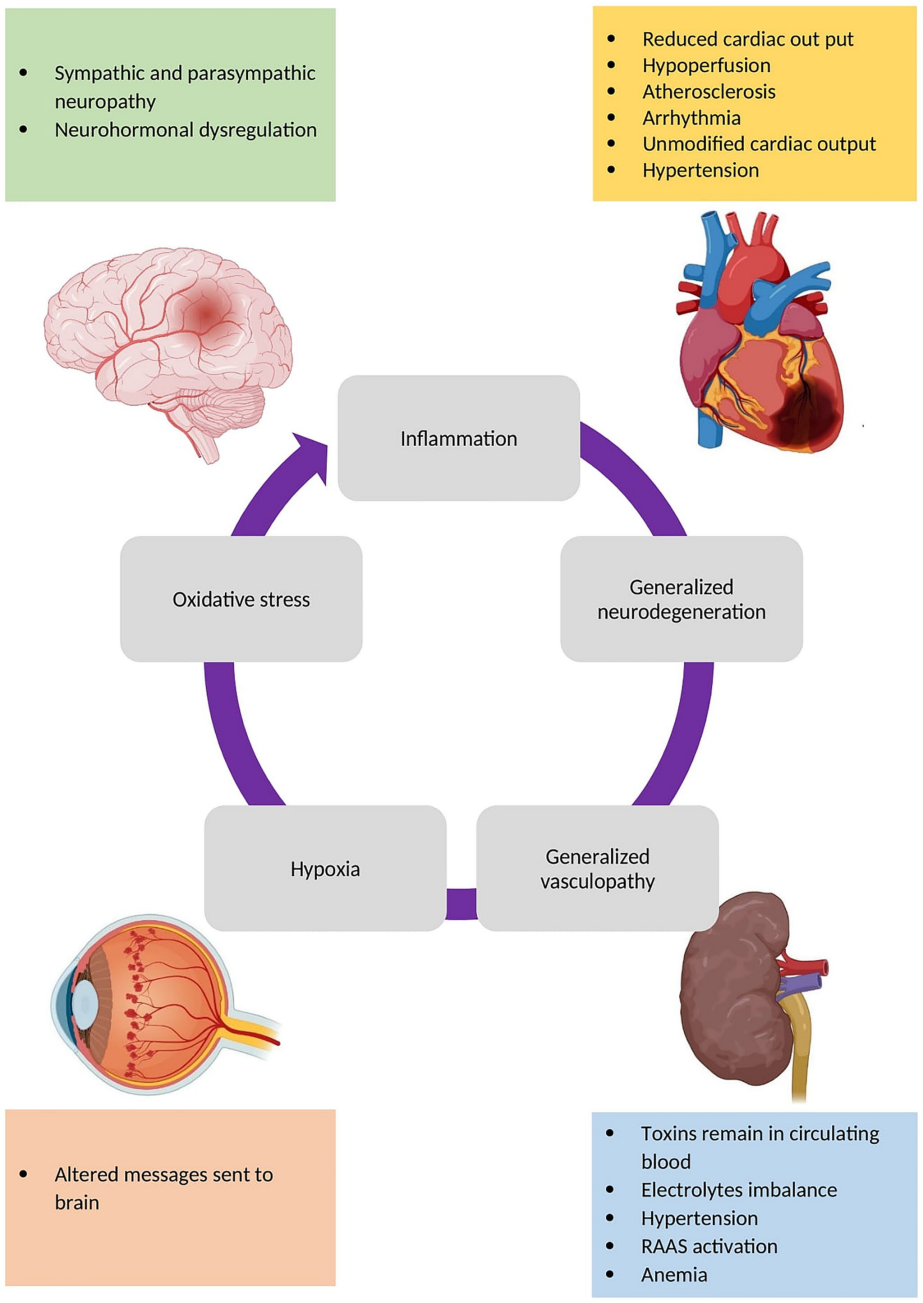
The mechanisms of different diabetes complications that affect other organs. Consequent to all of these mechanisms, inflammation, neurodegeneration, vasculopathy (such as angiogenesis and atherosclerosis), hypoxia, and oxidative stress occur in different organs leading to shared pathways discussed here.

Recent discoveries in the field of diabetes and its complications have demonstrated that diabetes complications are mutually reinforcing. For example, diabetic patients without nephropathy are at a lower risk of cardiovascular diseases, such as heart failure, than those with nephropathy. Interventional studies, such as randomized clinical trials, have demonstrated that the treatment of diabetic nephropathy can not only retard the progression of nephropathy but also decreases the risk of cardiovascular disease. Figure 2 summarizes the mechanisms by which different organs could impact each other (11, 12). Thus, an interdisciplinary approach could be beneficial for diagnosis and treatment of DM complications.

Fragmented care across multiple health care providers presents unique challenges for individuals with DM complications. Additionally, physicians may exhibit tunnel vision when managing a DM complication and fail to consider all complications, as well as their own specialty. Diverse clinical environments with healthcare facilities have the potential to enhance feasibility through interdisciplinary care. It is widely recognized that the clinical condition of diabetic patients is enhanced, and that complications and mortality rates are reduced during hospitalization when they are managed in an interdisciplinary manner (13, 14). Thus, a knowledge of shared pathological mechanisms among DM complications will be beneficial in an interdisciplinary approach for their diagnosis and treatments.

## 3 Shared pathological signaling mechanisms and cellular constituents

### 3.1 Vascular cell dysfunction

Vascular endothelial cells (EC), which line inside of the blood vessels, are likely the first type of cells to experience changes in systemic glucose levels. It is proposed that exposure of EC to high glucose results in their dysfunction, a condition in which the endothelium loses its ability to carry out essential physiological functions such as permeability, vasodilation, and regulation of coagulation and inflammation. These changes are extensively studied in the context of retinal vasculature both *in vitro* and *in vivo*, and are observed in individuals with DM. We now know that complications of DM such as diabetic retinopathy (15), diabetic nephropathy (16), diabetic neuropathy (17), and diabetic cardiomyopathy (18), are associated with vascular dysfunctions in these tissues. However, the detailed molecular and cellular mechanisms involved have not been well delineated. Hyperglycemia induces a series of intracellular events that ultimately result in vascular dysfunction, likely throughout the entire vascular system. Consequently, the identification and targeting of these shared pathological events could simultaneously impact all organs.

Vascular EC are highly differentiated and have limited proliferative capacity but can be activated by changes in the production of autocrine and/or paracrine angioinflammatory regulatory factors. The impact of diabetes on the production of these angioinflammatory regulatory factors could alter the EC viability and function leading to vascular dysfunction and degeneration (19–22). Our knowledge of these regulatory factors is limited and has been mainly focused on retinal vascular EC. We showed the exposure of mouse retinal EC to high glucose conditions results in the downregulation of a key angioinflammatory regulatory protein, namely thrombospondin-1 (TSP1) (23). This is concomitant with enhanced retinal vascular EC proinflammatory and proangiogenesis activities (24). In addition, the lack of TSP1 expression exacerbated the development and progression of diabetic retinopathy in preclinical mouse models of diabetes (21). Furthermore, we showed that TSP1 level is lower in vitreous humor samples from humans with diabetes compared with non-diabetes (25).

We also showed retinal EC exhibits a very low rate of apoptosis under normal glucose conditions (0.1–0.3%), which was not significantly affected when retinal EC was exposed to high glucose conditions (26). However, this could become significant after years of chronic hyperglycemia *in vivo* (27). In addition, we found increases in the extracellular glucose levels minimally impacted the intracellular glucose levels in retinal EC (22). Furthermore, given the tissue specificity of EC, it is unclear how EC from other organs responds to high glucose levels. Thus, the impact of high glucose conditions on retinal EC metabolic activity is minimal and will benefit from studies in EC from other organs impacted by diabetes (28).

The other major vascular cells targeted in DM are the pericytes (PC) whose recruitment and interaction with EC are vital to the integrity and function of blood vessels (20). Retinal vasculature has the highest ratio of PC/EC (1:1 in humans) than any other tissue. Dysfunction and loss of PC have a significant impact on retinal vasculature permeability and degeneration (29). Pericyte loss from retinal vasculature with diabetes has been recognized as one of the earliest signs of diabetic retinopathy. Determination of the underlying mechanisms has been the focus of many studies. We have found that retinal PC exhibits a 10-fold higher rate of apoptosis compared with retinal EC under normal glucose conditions (1–3%), and exposure of PC to increased extracellular glucose level results in a significant increase in their apoptosis rate (30). In addition, incubation of retinal PC under high glucose conditions, unlike retinal EC, resulted in significantly increased intracellular glucose levels. This was concomitant with enhanced activity of hexosamine biosynthesis activity and increased O-GlcNacylation of many proteins detected in retinal PC, including the P53 protein (30). O-GlcNacylated P53 is protected from phosphorylation and subsequent ubiquitination and proteasome degradation. The increased P53 level is consistent with enhanced apoptosis noted in PC incubated under high glucose conditions (31). Thus, the metabolic activity of retinal PC is significantly impacted by high glucose levels resulting in increased oxidative stress and inflammatory changes (28), and their apoptosis which contributes to the demise of EC and the degeneration of retinal vasculature (32).

Vascular damage including EC and PC loss could be repaired, at least in part, by circulating progenitor cells in the blood. In diabetes, the number of vascular progenitor cells becomes limited (33). Consequently, it is reasonable to target vascular dysfunction in DM complications. Extracellular stimuli activate various signaling pathways through specific receptors including integrins. Integrins stimulate p38- mitogen-activated protein kinase (MAPK) and Jun N-terminal kinase (JNK) activity, resulting in vascular cell death. The endoplasmic reticulum of EC could suppress the activation of VE-cadherin in response to DM chronic stress, resulting in caspase activation and apoptosis (34).

Two feasible options exist for vascular repair. First, mobilization of vascular progenitor cells from the bone marrow for vascular repair, and second, inhibition of the cell death pathways to preserve vascular integrity (35). The activation of the stromal cell-derived factor (SDF)-1α/ C-X-C chemokine receptor type 4 (CXCR4) axis appears to mobilize endothelial progenitor cells into the peripheral circulation. Direct targeting of cell death pathways is challenging because normal cells require these pathways to eliminate premalignant, damaged, and infected cells. However, activation of the SDF-1/CXCR4 axis does not carry this limitation. Vascular EC repair is currently treatable with available diabetes medications. In diabetic patients, Sitagliptin, a dipeptidyl peptidase (DPP)-4 inhibitor, and liraglutide, a GLP-1 receptor agonist, enhance the level of endothelial progenitor cells (36). Similarly, bone marrow-derived, or resident tissue mesenchymal stem cells could replenish the lost PC and stabilize the damaged blood vessels (37–39). Although these strategies have shown limited success, their validity and clinical translation will benefit from additional preclinical and clinical studies (40). Here we will further discuss shared intracellular signaling pathways with impact on the functional integrity of various organs affected by diabetes, whose targeting may have therapeutic benefits in multiple organs.

### 3.2 Bradykinin-1 receptor signaling pathway

The bradykinin-1 receptor is a component of the bradykinin/bradykinin-1 receptor pathway, which regulates vascular permeability and cytokine release (41). The Bradykinin-1 receptor plays a crucial role in various diseases. Occasionally, its inhibition is protective. In other instances, however, inhibiting this receptor can be deleterious. These findings demonstrate that the behavior and downstream effect of this receptor are context- and time-dependent (42, 43). The Bradykinin-1 receptor is strongly associated with tissue damage and inflammation. Based on *in vivo* investigations of DM, the expression of this receptor increases in multiple retinal layers and cell types just 2 weeks after the induction of diabetes in *in vivo* models (44, 45), whereas it is undetectable in normal retinal cells under physiological conditions (45).

In the retina, the Bradykinin-1 receptor can regulate vascular dilation and permeability. In the early stages of DM, blocking the bradykinin-1 receptor is associated with decreased leukostasis, hypoxia-inducible factor (HIF)-1α expression, vascular endothelial growth factor (VEGF) expression, and vascular permeability (45, 46). However, a bradykinin-1 receptor antagonist failed to improve clinical outcomes of diabetic macular edema (DME) and diabetic retinopathy in a human study (47). In another study using an *in vivo* model of DM, the bradykinin-1 receptor was protective 4 days after the onset of DM, but not after 6 weeks (43). A separate *in vivo* study revealed that pancreatic kallikrein, which can stimulate the bradykinin-1 receptor, is protective when administered immediately after the onset of DM (48, 49). Taken together, the clinical outcomes and protection afforded by bradykinin-1 receptor antagonism in the retina appear to be dependent on the duration of diabetes (43, 45).

Plasma levels of bradykinin rise in diabetics without clinical signs of diabetic nephropathy. Increased bradykinin appears to be a protective response for glomerular function (50). In the absence of the bradykinin-1 receptor, diabetic nephropathy is exacerbated (51). In subsequent stages, opposite results were observed. An antagonist of the bradykinin-1 receptor was administered one and 4 weeks after the induction of DM in an *in vivo* study. This pharmacological intervention decreased abnormally elevated vascular permeability in the renal medulla while normalizing microvascular permeability in the renal cortex (52). These are the early indicators of diabetic nephropathy. The impact of the bradykinin axis in the late stages of diabetic nephropathy will benefit from additional research, as is the case with diabetic retinopathy.

Bradykinin is a well-known mediator involved in inflammation and pain. In neurons, the bradykinin-1 receptor participates in the sensitization of sensory neurons (53). Neurons express bradykinin-1 receptors at extremely low levels (53, 54). This receptor is involved in intracellular calcium mobilization in neurons (54). The absence of this receptor at the onset of DM is linked to a worsening of diabetic neuropathy (51). However, further research has demonstrated that antagonizing the bradykinin-1 receptor 4 weeks after the onset of DM can reduce oxidative stress and restore neuronal function (55). In diabetic neuropathy, the bradykinin-1 receptor is also involved in hyperalgesia. Inhibition of this receptor reduces hyperalgesia (56). These results suggest that it is crucial to administer bradykinin-1 receptor antagonist at the right time. Improper inhibition of this receptor can have harmful or ineffective consequences.

The outcome of Bradykinin changes in the heart is similar to that of the retina, kidney, and peripheral neurons. One study demonstrated that increased activity of the kinin-kallikrein system, which includes the bradykinin-1 receptor, is protective for the heart in early-stage of DM (48). The suppression of bradykinin-1 receptor activity, on the other hand, reduces the increased vascular permeability in the heart during the later stages of DM (52). Few studies have examined the role of the bradykinin-1 receptor in diabetic cardiomyopathy. Thus, additional research is required to further clarify the role of the bradykinin axis in the development and progression of diabetic cardiomyopathy.

### 3.3 Wnt/*β*-catenin signaling pathway

Wnts are extracellular glycoproteins that mediate intracellular communication via their receptors. Wnt-stimulated intracellular messages regulate cell proliferation, survival, behavior, and fate. The Wnt/*β*-catenin pathway, also known as the canonical Wnt pathway, is the most studied, especially in the context of diabetic retinopathy (57). The presence of Wnts is required to prevent the degradation of *β*-catenin within the cell. Wnts cause accumulation and translocation of *β*-catenin into the nucleus to affect transcription of target genes involved in homeostasis, repair, and metabolism in many tissues (58, 59). Dysregulation of Wnt/*β*-catenin signaling is associated with numerous pathologies (58), including DM and its complications (60). Thus, delineating the roles of the Wnt/β-catenin pathways in these pathologies is essential for their diagnosis and treatment, and improved patient care.

The *β*-catenin expression and translocation to the nucleus are increased in diabetic retinopathy. Suppression of Wnt signaling decreases inflammation, blood-retinal barrier (BRB) dysfunction, and angiogenesis, which drives the development and progression of diabetic retinopathy (61). Oxidative stress is one of the primary stimuli for increased Wnt/*β*-catenin activity and inflammation (62). A monoclonal antibody targeting the Wnt receptor was tested in a preclinical investigation of diabetic retinopathy and demonstrated decreased VEGF levels and reduced expression of the adhesion molecules that mediate leukostasis and inflammation (63). Other strategies to mitigate Wnt/*β*-catenin signaling such as treatment with melatonin (64) and fenofibrate (65) also have shown promising results in halting the development and progression of diabetic retinopathy.

Wnt/β-catenin signaling is required for podocyte survival, differentiation, and motility in the kidney (66). In diabetic nephropathy, however, an overactive Wnt/*β*-catenin pathway causes podocyte dysfunction (67) and apoptosis (68) in the nephrons. The precise mechanisms involved remain largely unresolved. However, an abnormally high level of Wnt signaling inhibits the expression of nephrin, which is essential for the control of kidney filtration (67). Moreover, Wnt/*β*-catenin is involved in cross-talk between various cell types in diabetic nephropathy, which ultimately leads to proteinuria and abnormal filtration characteristics in diabetic nephrons (69).

Wnt/*β*-catenin signaling is essential in the process of neuronal myelination (70). This pathway also plays a role in neuropathic pain, which can be suppressed by pharmacological targeting (71). In diabetic peripheral neurons, the expression of Wnt/*β*-catenin signaling components increases (72). Furthermore, inhibiting the Wnt/*β*-catenin pathway can prevent diabetic peripheral neuropathy in rats (73).

Wnt/β-catenin is also involved in cardiac development. The expression of various members of this pathway decreases during maturity. In adulthood, however, low Wnt/*β*-catenin signaling activity is required to maintain normal mitochondrial function and metabolism (74). Nevertheless, increased activity of Wnt/β-catenin signaling is associated with cardiovascular pathologic changes such as hypertrophy and remodeling, which are mitigated by inhibition of this pathway (75). In DM, inhibiting Wnt/β-catenin signaling can reduce cardiac fibrosis (76).

### 3.4 Rho-associated kinase signaling pathway

ROCK regulates actin cytoskeleton organization and influences cell morphology, motility, polarization, phagocytosis, and gene expression with significant physiological functions. Moreover, its abnormal activity is implicated in the pathogenesis of a variety of diseases, including malignant and nonmalignant diseases (77). ROCK is highly activated in diabetic retinopathy (78), diabetic nephropathy (79), diabetic neuropathy (80), and diabetic cardiomyopathy (81), and perhaps impacted by the Wnt/*β*-catenin signaling axis.

In diabetic retinopathy ROCK activation alters the distribution of claudin-5 in the inner blood-retinal barrier (BRB) of the retina, resulting in BRB impairment and inflammation. Ripasudil, a ROCK inhibitor, can prevent both BRB impairment and inflammation in the retina (78). Moreover, ROCK activation constricts vessels leading to hypoxia. In addition, ROCK activation also alters the cytoskeleton of retinal pigment epithelium cells, compromising the outer BRB. Intravitreal injections of fasudil, a ROCK inhibitor, reduces retinal pigment epithelium shape alterations and retinal hypoxia (82). We have shown that fasudil alone (83) or in combination with bevacizumab (84) is effective in the treatment of DME in humans. Other ROCK inhibitors, such as AMA0428, are under investigation for the treatment of diabetic retinopathy (85). We also found that the ROCK inhibitor Y-27632 protects retinal PC from high glucose-mediated cell death in culture (31). As a common antidiabetic treatment, glucagon-like peptide (GLP)-1 receptor agonists can also inhibit ROCK. In *in vivo* models of diabetes, GLP-1 receptor agonists preserved the integrity of BRB by inhibiting ROCK (86).

ROCK activation is associated with the rise of HIF-1α in diabetic nephropathy (79). HIF-1α causes renal fibrosis (87). The inhibition of ROCK by fasudil slows the evolution of diabetic nephropathy (79) and improves renal hemodynamics in the *in vivo* models of DM (88). In addition, fasudil modulates the immune system to suppress inflammation by altering the polarization of macrophages, thereby decreasing renal injury in DM (89).

ROCK activation inhibits axonal regeneration (90) and is one of the driving forces behind diabetic neuropathy (91). A meta-analysis reported the improvement of therapeutic regimen efficacy in reducing neuropathic symptoms by the addition of fasudil to other diabetic neuropathy treatments (92). ROCK activation also results in vascular EC dysfunction, contraction of vascular smooth muscle cells, and myocardial fibrosis (81, 93). It is well-recognized that the heart of a diabetic patient has elevated aberrant ROCK activity (81). A study on the heart of diabetic rats has revealed that fasudil improves calcium homeostasis and remodeling of cytoskeletal structure (94).

### 3.5 Notch signaling pathway

Notch receptors in vasculature are mainly expressed in arterial blood vessels. Delta-like 4 (Dll4) is the ligand of Notch receptors and promotes arterial blood vessel branching via EC sprouting (95). Notch receptors are not only present in vessels but are also expressed on the surface of macrophages and other immune cells. Overactivation of Notch receptors result in inflammation contributing to numerous pathologies including DM complications (96). The expression of Dll4 and Notch receptors, as key players in the pathogenesis of diabetic retinopathy, is increased in the diabetic retina leading to angiogenesis (97).

Increased Notch receptor activity correlates with macrophage activity. When macrophage activity increases, the release of inflammatory mediators is increased. This results in an inflammatory environment within the diabetic kidney (96). Hyperglycemia increases Notch signaling activity. Notch signaling increases VEGF expression. These alterations ultimately lead to podocyte loss in the kidney (98). In addition, excessive Notch signaling induces caspase-dependent apoptosis of podocytes via the mitochondrial pathway (99).

Another complication of DM associated with Notch signaling is diabetic neuropathy. Notch signaling pathways may play a role in the induction and maintenance of neuropathic pain through three mechanisms. These include activation of glial cells, enhancement of synaptic transmission, and alteration of ion channels (100). Inhibition of Notch signaling can reduce neurodegeneration and halt the progression of diabetic neuropathy in a rat model of peripheral neuropathy (94, 101, 102).

The information regarding Notch signaling in diabetic cardiomyopathy is controversial. According to a study on diabetes (58), increased Notch signaling induces a fibrotic response in the heart, and Notch inhibition can diminish diabetic cardiac remodeling (103). However, in another study, increased Notch activation decreased apoptosis in the diabetic heart (104). Thus, more studies are needed to resolve these discrepancies.

### 3.6 Complement 3a signaling pathway

Complement activation is one of the characteristics shared among DM complications and appears to be one of the mechanisms underlying angiogenesis and vascular dysfunction (105). As a component of the complement system, C3a plays a crucial role in pathophysiology of the DM complications. C3a, depending on the circumstances, is a pro- or anti-inflammatory mediator and is associated with an increased risk of diabetic retinopathy, nephropathy, neuropathy (106), and cardiomyopathy (107).

C3a levels are elevated in the vitreous samples from patients with proliferative vitro retinopathy (PVR) (108, 109). However, the sources and effects of C3a remain largely unresolved. It appears that retinal PC secrete autoantibodies that could activate the complement system. One of these complements is C3a, which causes PC loss (110). In diabetic retinopathy, the therapeutic effect of C3a inhibition has not been evaluated thus far. The inhibition of C3, however, reduces neurodegeneration in age-related macular degeneration (111).

In DM, C3a induces inflammation and an adaptive immune response that damages the kidney. As a key component in the pathophysiology of diabetic nephropathy, C3a also increases cytokine secretion from macrophages enhancing inflammation. In diabetes, the absence of the C3a receptor reduces inflammation and renal damage (112). In diabetic nephropathy, C3a also participates in mitochondrial fragmentation and reduced antioxidant capacity of podocytes. Blocking the C3a receptor reduces oxidative stress and podocyte damage (113). Thus, targeting C3a or its receptor seems a logical treatment option for diabetic nephropathy.

Extensive research on the C3a’s role in the pathophysiology of diabetic neuropathy is lacking. However, it is known that increased C3a levels are associated with a higher risk of diabetic neuropathy (106). Activation of other components of the complement system impairs the blood supply to endoneurium, the connective tissue surrounding myelinated nerve fiber layers. Consequently, it is possible that C3a compromises the blood supply to protective tissues surrounding peripheral nerves (114). However, the direct effects of C3a in diabetic neuropathy are an intriguing subject for future research.

C3 is primarily generated in the liver and adipose tissues. C3 activation results in the production of C3a. Thus, obesity as a risk factor for DM and cardiovascular diseases (76) partially explains the elevated C3a levels in these conditions. Although increased C3a has not been conclusively linked to diabetes-induced cardiomyopathy, its level increases independently in diabetes and cardiovascular disorders. Further research is needed to clarify the contribution of C3a to diabetic cardiomyopathy.

### 3.7 Leucine-rich α2-glycoprotein 1 and transforming growth factor *β* signaling pathways

Numerous tissues, including the kidney, heart, and nervous system produce LRG1. This inflammatory biomarker is involved in numerous physiological processes including synaptogenesis, neurotransmitter release, cell–cell adhesion, and protein–protein interactions. Additional investigations have uncovered the pathologic function of LRG1 in a wide variety of diseases. TGF-*β* primarily drives the pathological function of LRG1 (77, 78). LRG1 and TGF-β can induce neovascularization and play a role in respiratory diseases, cancer, and endocrine diseases such as DM and its complications (115, 116).

Under pathological conditions such as DM, LRG1 is recognized as a promoter of neovascularization and vascular dysfunction (117). LRG1 induces angiogenesis via TGF-*β* during the development and progression of diabetic retinopathy (118). Its blood and vitreous levels increase with the progression of diabetic retinopathy to the proliferative stage (119). The role of TGF-β in angiogenesis associated with the progression of diabetic retinopathy is well established. Thus, targeting TGF-β or LRG1 may have a positive impact in halting the development and progression of diabetic retinopathy.

In the kidney, LRG1 is primarily localized in renal glomeruli and its level rises in diabetic nephropathy. The elevated levels of LRG1 in diabetic nephropathy are associated with glomerular neovascularization and podocyte loss (120). An intriguing aspect of LRG1 in diabetic nephropathy is that LRG1 expression rises before VEGF levels, and targeting LRG1 may be a logical step in preventing diabetic nephropathy before significant kidney damage occurs (121). Metformin inhibits the angiogenesis induced by changes in LRG1 and TGF-*β* levels in diabetic kidney (122).

Research on LRG1 in diabetic neuropathy remains very limited. However, studies conducted in preclinical models of DM demonstrated that LRG1 could serve as a promising target for the treatment of diabetic erectile dysfunction, a complication associated with neuropathy and microangiopathy (85). Further research is needed to unravel the consequences of changes in LRG1 during the development and progression of diabetic neuropathy.

The higher level of LRG1 in blood is associated with an increased risk of heart failure in diabetic patients (123). In addition, changes in the LRG1 and TGF-*β* levels contribute to cardiac remodeling (124). Increased levels of LRG1 are shown to contribute to endothelial cell dysfunction and arterial rigidity in diabetic patients (125). Thus, targeting LRG1 and TGF-β may also have beneficial effects in the treatment of diabetic cardiomyopathy.

### 3.8 Doublecortin-like kinase 1 signaling pathway

DCLK1 is a member of the microtubule-related protein family and an overlooked pro-oncogenic gene. DCLK1 can regulate its kinase activity to avoid unnecessary phosphorylation in its microtubule-binding domain (126). This is crucial for the role of DCLK1 in promoting neuronal survival following injury (127) and regulating dendritic growth and synaptic maturation (128). DCLK1 also triggers NF-κB activation, leading to the initiation of inflammatory responses in cardiac cells (128, 129). The majority of our understanding regarding DCLK1 function is confined to its role in cancer. However, there are some studies investigating the role of DCLK1 expression in DM complications.

A recent *in vivo* study revealed that the expression of DCLK1 significantly rises in the cardiomyocytes of diabetic hearts. DCLK1 plays a crucial role in controlling the inflammatory responses associated with diabetic cardiomyopathy. Blocking DCLK1 action resulted in decreased cardiac fibrosis and hypertrophy, as well as a reduction in NF-κB activity (129). Inflammation-induced by DCLK1 also plays a role in atherosclerosis (130). Additionally, a separate study revealed that DCLK1 plays a role in obesity-induced cardiomyopathy, indicating that its impact extends beyond DM (131).

DCLK1 expression increases in individuals with diabetic nephropathy (132). The specific role DCLK1 plays in diabetic nephropathy and neuropathy has not been thoroughly investigated. Cancer research has shown that DCLK1 plays a role in regulating Notch signaling activity (133) which is involved in DM complications such as retinopathy, nephropathy, neuropathy, and cardiomyopathy (96, 103). Further research on DCLK1’s roles in the development and progression of diabetes should provide valuable insight into a more targeted management of DM complications.

### 3.9 Eukaryotic initiation factor 4E signaling pathway

EIF4E is a crucial protein involved in mRNA cap-binding, playing an essential part in mRNA-ribosome interactions and translation in eukaryotic cells. EIF4E plays a crucial role in facilitating the transfer of mRNA to the ribosome and is essential for the initiation of translation. When it comes to DM, studies have demonstrated the crucial role of EIF4E in insulin secretion and maintaining proper glucose levels (134, 135). In addition, increased levels of EIF4E are linked to a reduced risk of DM (136). The interaction of EIF4E with other molecules, such as 4E-BP1, plays a crucial role in development of the DM complications (137).

In diabetic retinopathy, there is a correlation between elevated 4E-BP1 binding to EIF4E and upregulation of VEGF expression (137, 138). Additionally, this binding is linked to oxidative stress and impaired mitochondrial function (139). Currently, there is no research available focusing on EIF4E in diabetic retinopathy. However, this topic has been studied in the context of diabetic nephropathy. Dephosphorylation of EIF4E is proposed as a potential mediator of fibrosis in diabetic nephropathy (140). Pirfenidone dephosphorylates EIF4E, protecting the kidneys in DM (141). However, in the heart of diabetic patients, the activity of EIF4E decreases resulting in a limitation of protein synthesis that ultimately contributes to diabetic cardiomyopathy. There are various methods to focus on EIF4E activity that have not been thoroughly examined in relation to DM complications. Further research could provide valuable insight into the practical aspects of this molecule and its gene expression (142).

### 3.10 MFF (mitochondrial fission factor) signaling pathway

The mitochondrial dynamic consists of two main components: fission and fusion. Mitochondrial fission involves the formation of a ring by DRP1 around the mitochondria, resulting in its division into two daughter mitochondria. Receptors are required for DRP1 to attach to the outer membrane of mitochondria. MFF is one of these receptors (143), and its expression increases in the diabetic retina (144). A study found that melatonin has the ability to reduce MFF expression in the diabetic retina. This reduction helps to maintain the integrity of the BRB and with a positive impact on mitochondrial function (145).

There is an ongoing debate surrounding our understanding of the MFF’s role in diabetic nephropathy. Some studies have indicated that MFF plays a role in mitochondrial fission, oxidative stress, and the release of proapoptotic molecules from mitochondria in diabetic nephropathy (146, 147). On the other hand, a different study showed that MFF protects against injury to podocytes caused by hyperglycemia (148).

The lumbar dorsal root ganglion of diabetic mice similarly exhibits increased MFF expression (149). However, there is a lack of research on potentially targeting MFF in diabetic neuropathy. MFF is also implicated in the development of atherosclerosis. Metformin decreases the development of atherosclerosis in individuals with DM by preventing the interaction between DRP1 and MFF (150). In contrast, MFF expression decreases in the heart of individuals with DM. Empagliflozin increases MFF expression in the heart (151). Further research is necessary to investigate the impact of MFF on DM complications and the development of innovative approaches to regulate MFF expression.

### 3.11 Growth-associated protein 43 signaling pathway

GAP43, a gene specific to neural tissues, is implicated in neural plasticity, regeneration, and development in the peripheral and central nervous systems (152). GAP43 serves as an indicator of axon health and regeneration. GAP43 levels decrease in the diabetic retina. A recent human study revealed a significant link between genetic variations in GAP43 and the development of diabetic retinopathy and nephropathy (153). Levetiracetam treatment has shown promising results in boosting GAP43 expression in the diabetic retina (154).

Non-diabetic animal models show that the absence of GAP43 is linked to cardiac hypertrophy and remodeling (155). In the context of a diabetic heart, there is a noticeable decrease in the expression of GAP43 in both myocardium and ganglion cells that innervate the heart (156). GAP43 increases in cardiac nerves (157) but in the diabetic heart, the elevated GAP43 levels may only be transient (158). Perhaps during the early stages of diabetes, an increase in GAP43 expression acts as a protective response to the adverse effects of diabetes on neurons (159). However, this response may change as DM persists. There is a decrease in GAP43 expression in the later stages of DM, which ultimately results in neurodegeneration. Prior to neuron loss in diabetic patients, decreased GAP43 levels are detected in diabetic neuropathy (160). Further research is required to investigate the impact of GAP43 on the development of DM complications and how it varies during various stages of DM.

## 4 Clinical and translational aspects of shared signaling pathways

### 4.1 Treatment strategies

**Figure 3:**
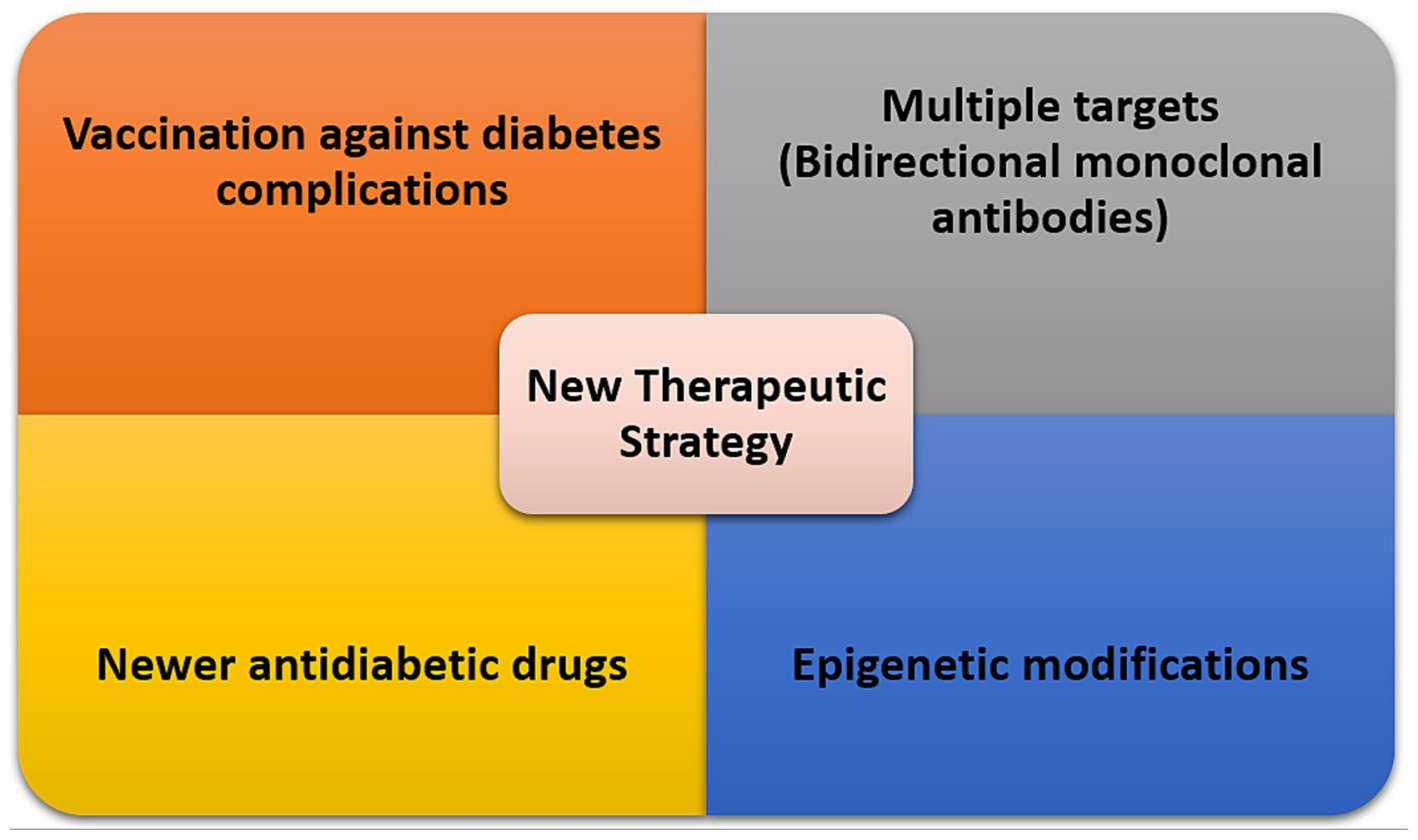
Therapeutic strategies for management of DM complications based on shared pathways.

Despite many efforts toward delineating the underlying mechanisms contributing to various complications of diabetes in different organs individually existing treatments do not reverse or prevent the progression of these complications. However, high glucose-mediated metabolic abnormalities appear to be key, and any efforts toward normalizing glucose levels will be beneficial systematically (161–163). Recent advancements in various genomic, transcriptomic, and proteomic strategies and their advancements to single-cell levels are beginning to provide significant details regarding a systems view of the changes associated with diabetes in different organs and their cellular constituents. The ability to examine multiple organs simultaneously for changes brought on by diabetes will allow a more global approach to the systemic ill effect of diabetes (164), as well as targeting shared and upstream pathways for effective treatment intentions. The investigation of non-coding RNAs including microRNAs and lncRNAs and their systemic circulation through small extracellular vesicles/exosomes are providing additional clues towards the identification of regulatory molecules targeted by diabetes that contribute to the pathogenesis of DM complications (165–171). We next discuss some areas of consideration relevant to the development of effective therapies for DM complications (Figure 3).

#### 4.1.1 Designing vaccines for DM complications

Subsequent to the global success in eliminating fatal infectious diseases, researchers have proposed the development of vaccines for non-infectious conditions, including complications of diabetes. This novel concept proposes that vaccines may be implemented in replacement of passive treatments with monoclonal antibodies. The human body is able to produce antibodies against a molecule that is overproduced in pathologic conditions as a result of vaccination (172). The potential to develop a vaccine for all DM complications may be facilitated by the existence of shared pathways. It has been demonstrated in a recent *in vivo* study that early vaccination against prorenin can prevent the development of early retinal lesions in diabetic models (173). In hypertensive patients and animal models of hypertension, vaccination against the renin-angiotensin system has been demonstrated to decrease systolic and diastolic blood pressure (174, 175). In an *in vivo* study of diabetic nephropathy, the progression of nephropathy and renal fibrosis was mitigated by vaccination against the renin-angiotensin system (176). Other targets that are currently being investigated to treat or prevent complications of diabetes include vaccination against Il-1β (177), Monocyte chemoattractant protein 1 (178), and AGEs (179).

Following the promising results of recent anti-diabetic medications like DPP-4 inhibitors and GLP-1 receptor agonists, researchers are now exploring the potential of DPP-4 and GLP-1 in developing novel vaccines (180). The impact of vaccination on complications related to DM has not been thoroughly examined. Nevertheless, studies have demonstrated that vaccination against DPP-4 has successfully postponed the development of DM in animal models (181). DPP-4 inhibitors have not shown the same level of cardiovascular and renal protection as GLP-1 receptor agonists and SGLT-2 inhibitors, which are considered more effective anti-diabetic drugs. As a result, recent studies exploring alternative pathways like SGLT-2 inhibition have shown even more encouraging findings (182). When it comes to addressing multiple complications simultaneously, there has not been any research conducted on the effectiveness of vaccinating against all diabetic complications. Further investigation into vaccination for common pathways in diabetic complications could potentially lead to the development of both therapeutic and preventive vaccines for managing these complications.

#### 4.1.2 Consideration of drug delivery, targeted therapy, optimal treatment time, and side effects

The proper distribution of medications is an issue of utmost significance in the treatment of DM complications, which should benefit from the mechanistic studies outlined above. For instance, a drug may reach the kidney, heart, and peripheral neurons through the body’s normal circulation. Systemic BRB, which separates the retina from the systemic circulation poses a challenge for the drug administration to the retina. Furthermore, the damaged circulation in the advanced phases of DM may contribute to the difficulties in drug delivery. Thus, optimizing drug delivery methods is necessary for effective treatment strategies (183).

Targeted therapy is an additional challenge in the treatment of DM complications. For instance, fasudil is one of the medications that inhibit several kinases including ROCK. Its systemic complications associated with its off-target effects could limit its therapeutic utility. In addition, some of the noted changes may have tissue and cell type-specific consequences. Thus, a clear understanding of the targeted pathways in various organs, tissues, and cellular constituents requires careful consideration in order to overcome the treatment’s systemic adverse effects (184).

The effectiveness and safety of interventions also depend on the DM stage. As previously mentioned, bradykinin-1 receptor antagonists can be ineffective, detrimental, or protective based on the stage of DM (45, 47). Thus, the design of a schedule that determines the optimum time for prescribing medication is needed to target any of the DM complications and will benefit from the development of early detection modalities.

Many of the therapeutic targets described in this review have normal physiological functions and their disruption could have adverse impacts. For example, LRG1 is involved in the formation and growth of synapses. However, its aberrant activity is linked to numerous diseases, including DM and its complications (185). Our grasp of the adverse effects associated with systemic administration of many treatments, which target pathways implicated in both physiological and pathological processes, is very limited. The majority of studies have concentrated on individual organs, overlooking their interconnections with one another. Hence, a more comprehensive and systemic evaluation is necessary after administering these types of treatments.

#### 4.1.3 Newer class of anti-diabetic drugs

Emphasis is given to the efficacy of newer categories of anti-diabetic drugs in managing DM complications. DPP-4 inhibitors such as sitagliptin and GLP-1 receptor agonists are capable of targeting some of the shared pathologies discussed. The efficacy of these treatments for DM complications is not simply limited to their ability to lower glucose levels. It is possible that these treatments have more therapeutic effects than the glucose-lowering properties, including anti-inflammatory and immune modulatory functions (186). In this regard, it is well established that these medications reduce the risk of diabetic renal and cardiovascular complications (187–189). Currently, the effects of semaglutide, a GLP-1 receptor agonist, on the progression of diabetic retinopathy is under investigation in the FOCUS trial (NCT03811561).

#### 4.1.4 Targeting metabolic memory

The concept of metabolic memory arises from the observation of patients who receive early treatment for DM but still experience DM complications despite maintaining appropriate glucose control. It’s clear that the pathophysiology of DM complications extends beyond just hyperglycemia. It appears that DM triggers inflammation, oxidative stress, and alterations in mitochondrial function. These pathological pathways interact with each other, influencing DNA methylation and manipulating epigenetics. As a result, the negative impact of high blood sugar will persist even after managing glucose levels. Ongoing research is being conducted to address metabolic memory and its potential to prevent complications of DM. Researchers are making efforts to target metabolic memory due to its dynamic and reversible nature, allowing for adjustments. These interventions encompass a range of approaches, including behavioral interventions, natural products, and targeted drugs (190).

#### 4.1.5 Targeting multiple pathways simultaneously

Extensive research has been dedicated to developing drugs that specifically target a single pathway involved in a disease. For example, over the past three decades, monoclonal antibodies have had a significant impact on the treatment of various diseases, including diabetic macular edema. Recent studies have shown that using newer monoclonal antibodies that target two molecules instead of just one could provide better outcomes. As anti-VEGF monoclonal antibodies, drugs like bevacizumab, ranibizumab, and aflibercept have made significant advancements in the treatment of diabetic macular edema (46). Recently, faricimab was introduced as a monoclonal antibody that simultaneously targets angiopoietin-2 and VEGF. A recent study found that faricimab, a treatment that targets multiple pathways, has shown better results compared to older treatments like ranibizumab and bevacizumab, which only target a single pathway (191). A method like this is crucial for complex diseases like DM and its associated complications. Prior studies on multi-target treatments have been stopped due to an extensive list of adverse effects associated with these therapies. Nevertheless, the promising outcomes of recent studies, such as the use of faricimab in treating diabetic retinopathy, provide a strong foundation for further research in this area (191, 192). By focusing on numerous shared pathways between DM complications, it may be possible to develop more effective treatments or even preventative measures.

### 4.2 Future studies based on shared pathophysiology

**Figure 4:**
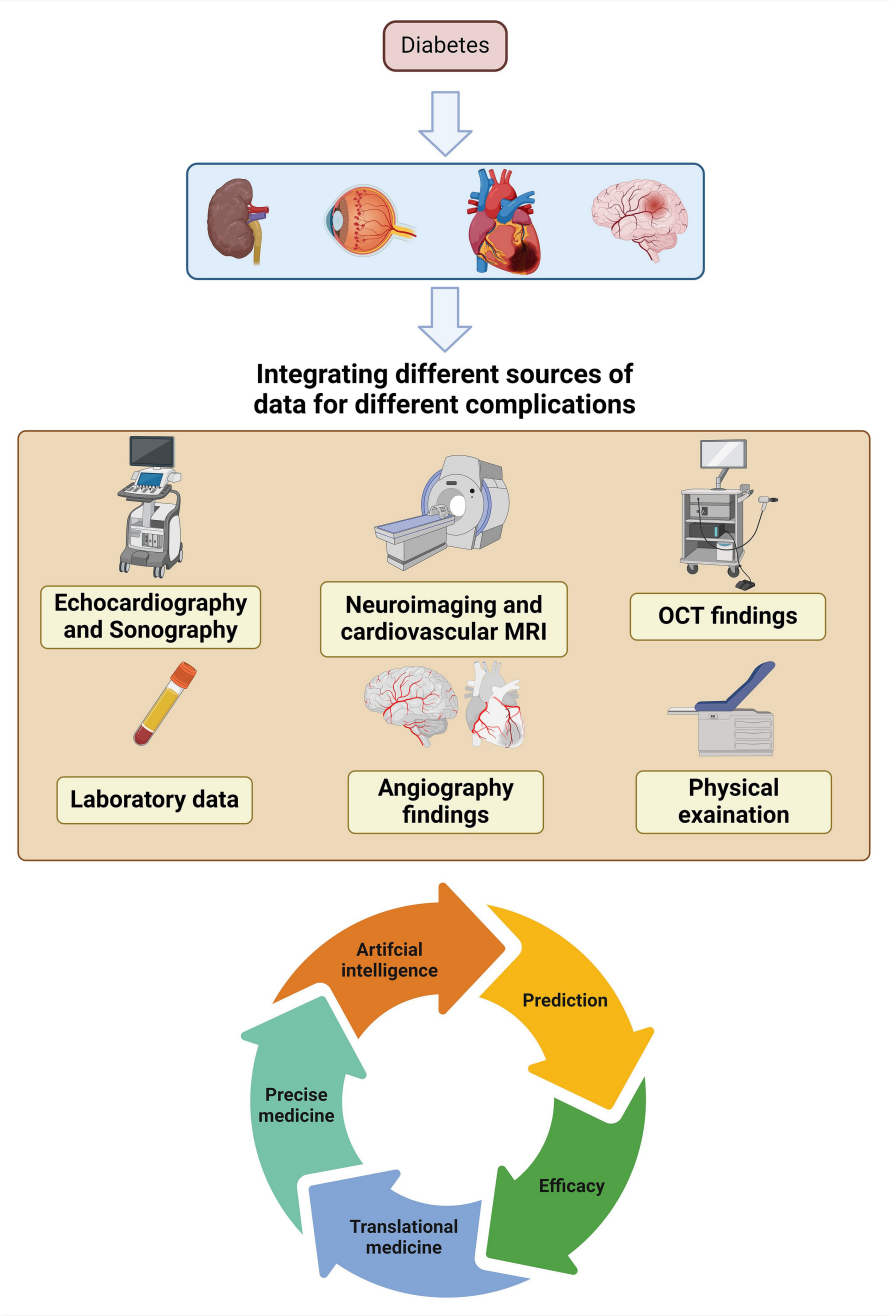
Pathway of future interdisciplinary studies of DM complications based on shared pathways.

As demonstrated in this paper, complications of DM exhibit various molecular pathways that might exacerbate one another. Therefore, future research can adopt a more comprehensive approach than before. As an example, the input data should not be restricted to a single complication. Additional data pertaining to various organs obtained from angiography, optical coherence tomography, sonography, echocardiography, laboratory data, and physical examinations can be combined and subjected to statistical analysis. Having such vast data allows us utilizing the full capabilities of artificial intelligence in DM research. Subsequently, we shall undergo further advancements in the fields of precise medicine, translational medicine, screening, and preventive. Furthermore, the incorporation of data in coming studies could potentially provide further insights into the unidentified pathophysiology of issues associated with DM (Figure 4).

**Figure 5:**
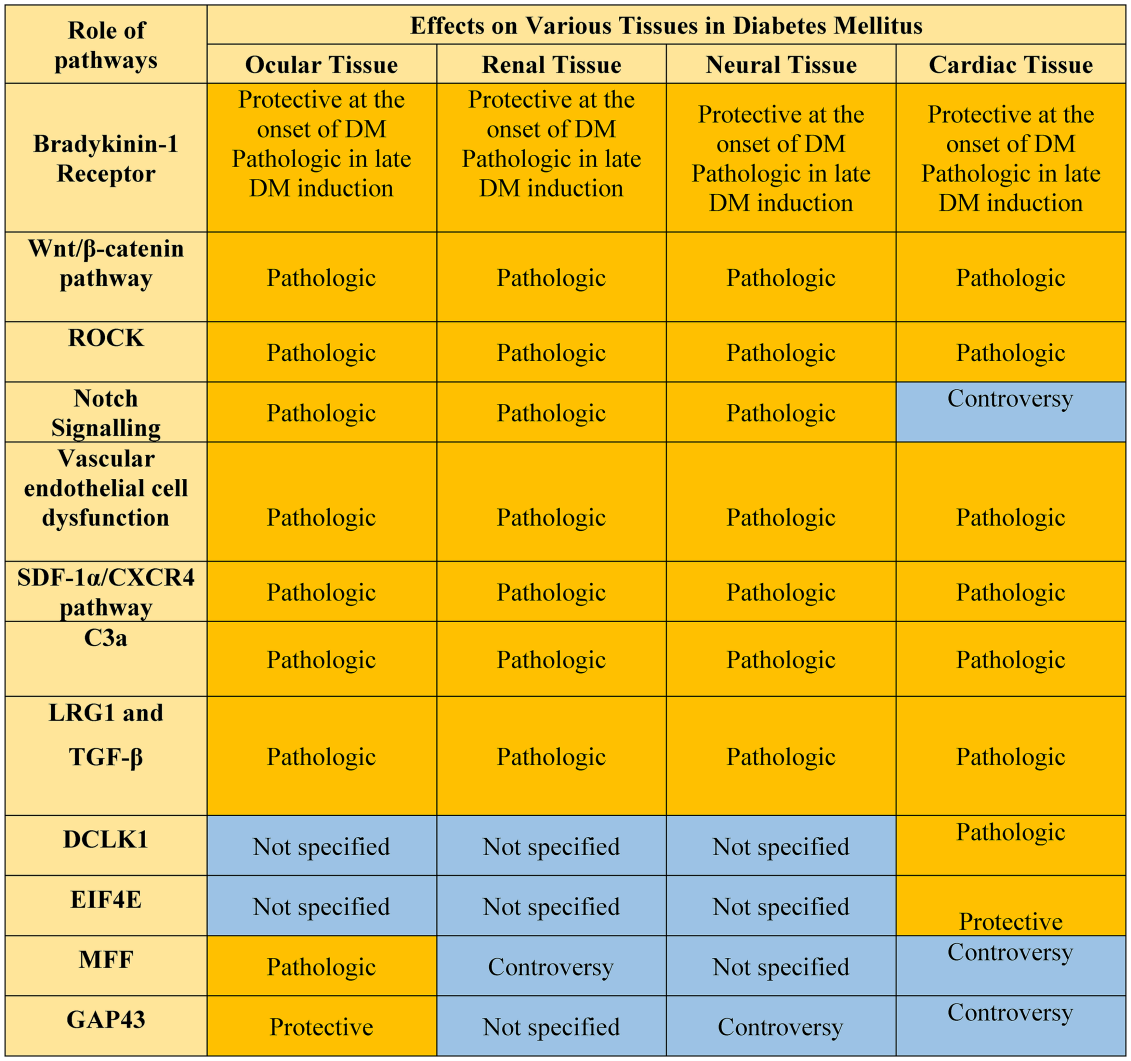
Common pathways in DM complications discussed in the paper. Those pathways with well-established roles are highlighted in yellow. Those pathways that need more studies are highlighted in blue.

In summary, over the past two decades, we have gained significant knowledge regarding the role of various molecular and cellular changes that contribute to the development and progression of DM complications in various organs and their cellular constituents (Figure 5). We also know more about the intercellular signaling pathways impacted during diabetes, some of which are shared among the DM complications, and some are tissue specific. However, the lack of suitable strategies for the prevention and treatment of DM complications necessitates a more systems view of the biochemical and cellular pathways that are impacted by DM. This requires a better understanding of the cellular constituents impacted, and the hierarchy and interactions among the various pathways identified in different DM targets. The recent advances in single-cell and tissue spatial RNA sequencing techniques examining all the target organs simultaneously could provide a broader understanding of unique and common pathways contributing to the development and progression of DM complications, especially at the early stages of the disease. This knowledge will aid in the development of a more comprehensive treatment, which addresses the impact of DM on all organs simultaneously. Furthermore, careful consideration of treatment strategies is also vital and deserves further consideration for the development of more comprehensive therapeutics for DM.

## 5 Conclusion

Our understanding of the pathophysiology of DM complications has extensively developed as these complications have been and continue to be separately studied. New studies using advanced multi-omics strategies, in the whole organ and its cellular constituents, will further aid in defining key early changes contributing to all DM complications. Existing research has already revealed a possible link among some of the DM complications that will be further advanced by systems-based designed studies. Thus, there are common pathways that could be identified and carefully targeted for the treatment of DM complications as a whole. By utilizing this approach, it is possible to improve the effectiveness of treatment and preventive measures for individuals with DM in the early stages. This area has been neglected for quite some time and will benefit from the development of new strategies for early non-invasive detection of DM complications and their more effective therapeutic targeting addressing the impact of a system as a whole.
